# Driveline Sepsis Presenting As Gout

**DOI:** 10.7759/cureus.7196

**Published:** 2020-03-06

**Authors:** Brittney Toms

**Affiliations:** 1 Nursing, Cardiac/Thoracic/Vascular Surgery, Hunter Holmes McGuire Veterans Affairs Medical Center, Richmond, USA

**Keywords:** left ventricular assist device (lvad), gout, septic arthritis, sepsis, heart failure, mechanical circulatory support (mcs), arthritis

## Abstract

In patients with a history of gout, there could be a delay in diagnosis of a septic joint, which increases morbidity and mortality. The literature reports rare instances of coexistent gout and septic arthritis. We present a 64-year-old male with non-ischemic cardiomyopathy, supported by a HeartWare ventricular assist device, who developed a methicillin-resistant Staphylococcus aureus (MRSA) driveline infection four months after device implant. He achieved suppression with minocycline 100 mg by mouth twice a day for five months before presenting to the emergency room with symptoms of gout. Joint aspirate was consistent with a diagnosis of MRSA as well as gout. The patient presented with typical symptoms of a percutaneous driveline infection: soreness at the exit site, erythema, and thick, purulent drainage. Wound culture of the driveline confirmed MRSA and guided antibiotic treatment. His presentation was unusual in that sepsis was identified only after he presented with septic arthritis, which led to the collection of blood cultures. He had no fever, chills, nausea, vomiting, or hypotension. This case illustrates that unresolving gout symptoms after one treatment, in a patient with a known driveline infection, should be further evaluated for possible sepsis and septic arthritis. The patient’s unusual presentation of sepsis caused difficulties in diagnosis and management. To our knowledge, this is the first reported case of a driveline infection seeding a joint and causing septic arthritis.

## Introduction

Driveline infections are the major cause of morbidity and mortality in ventricular assist device (VAD) patients [1]. Infections can remain limited to the driveline exit site, spread to the pump, or infect surrounding tissues [2]. To prevent severe complications, driveline infections must be promptly recognized and appropriately treated. Driveline infections are typically treated with oral or intravenous (IV) antibiotics for a period of time as recommended by the infectious disease team and then transitioned to suppressive antimicrobial therapy with oral agents as appropriate [2]. When antibiotics fail to treat the localized infection, patients may become bacteremic and experience septicemia. Bacteremia also has the potential to seed gouty joints leading to septic arthritis. In patients with a history of gout, it can be easy to misdiagnose a septic joint, which delays care and increases morbidity and mortality [3,4]. The literature reports rare instances of coexistent gout and septic arthritis. However, to our knowledge there are no known cases of acute driveline sepsis appearing in such a manner.

## Case presentation

**Figure 1:**
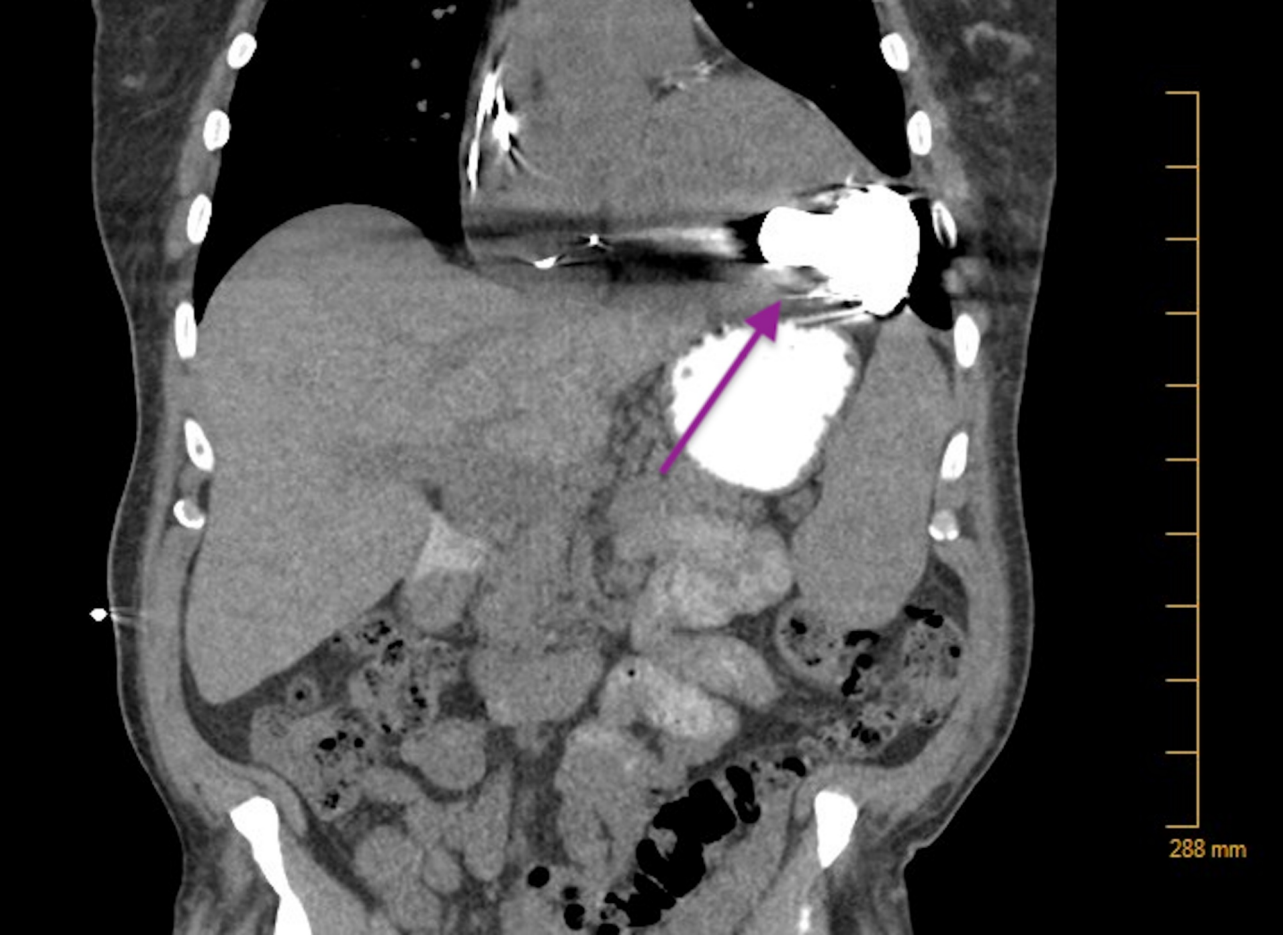
CT of the Thorax No fluid collection noted around the pump.

**Figure 2:**
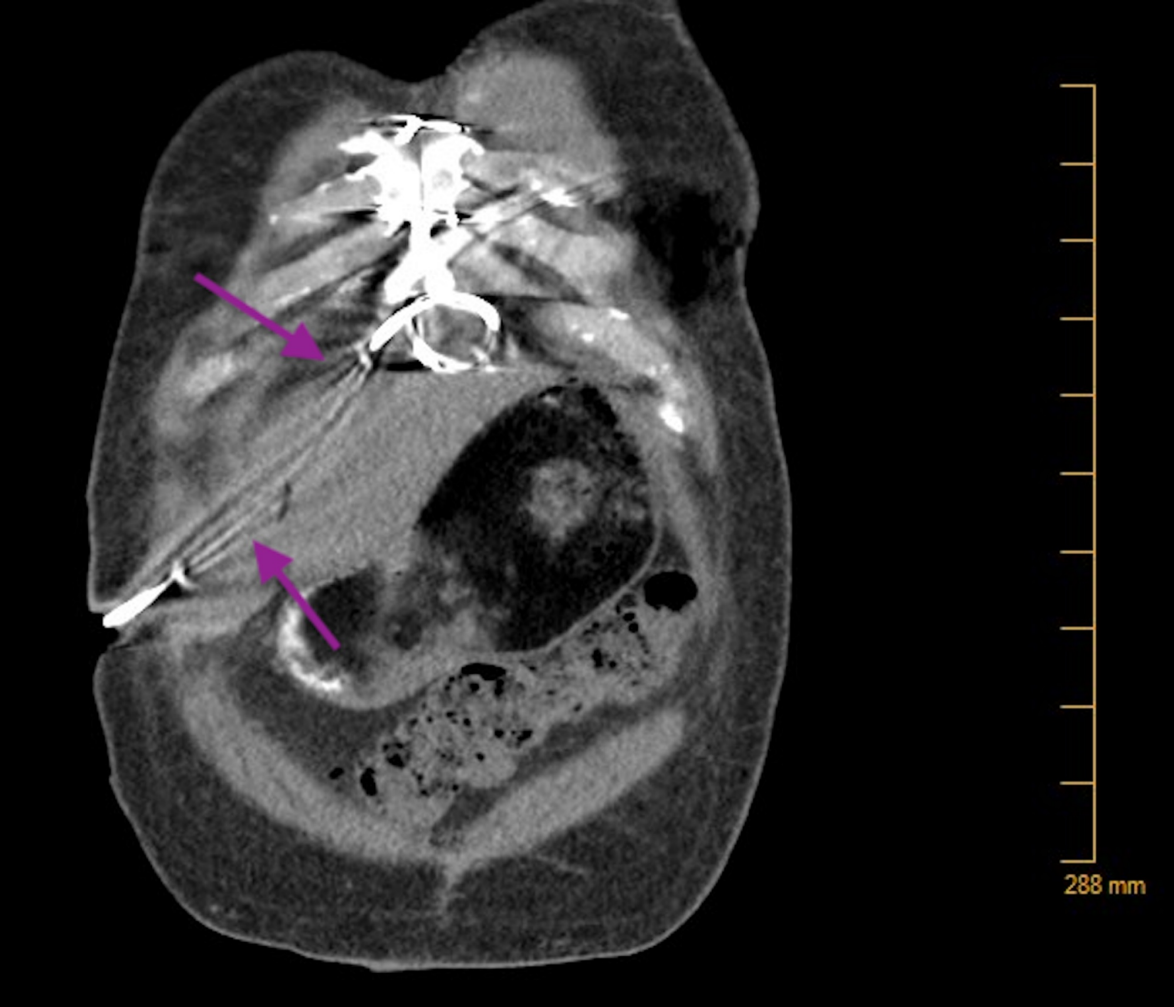
CT Scan of the Abdomen and Pelvis No fluid collection noted along the driveline.

A 64-year-old African-American male with non-ischemic cardiomyopathy managed with a HeartWare VAD presented to an outside emergency room (ER) via wheelchair with complaints of left knee pain for 12 days. Past medical history was significant for gout, hypothyroidism, blood dyscrasia, and chronic kidney disease stage III. He was diagnosed with a driveline infection localized to the percutaneous tissue four months ago. At the time of diagnosis, the sensitivity report guided antibiotic choice. A computerized tomography (CT) scan confirmed infection localized to the abdominal tissue (Figures 1, 2).

**Figure 3:**
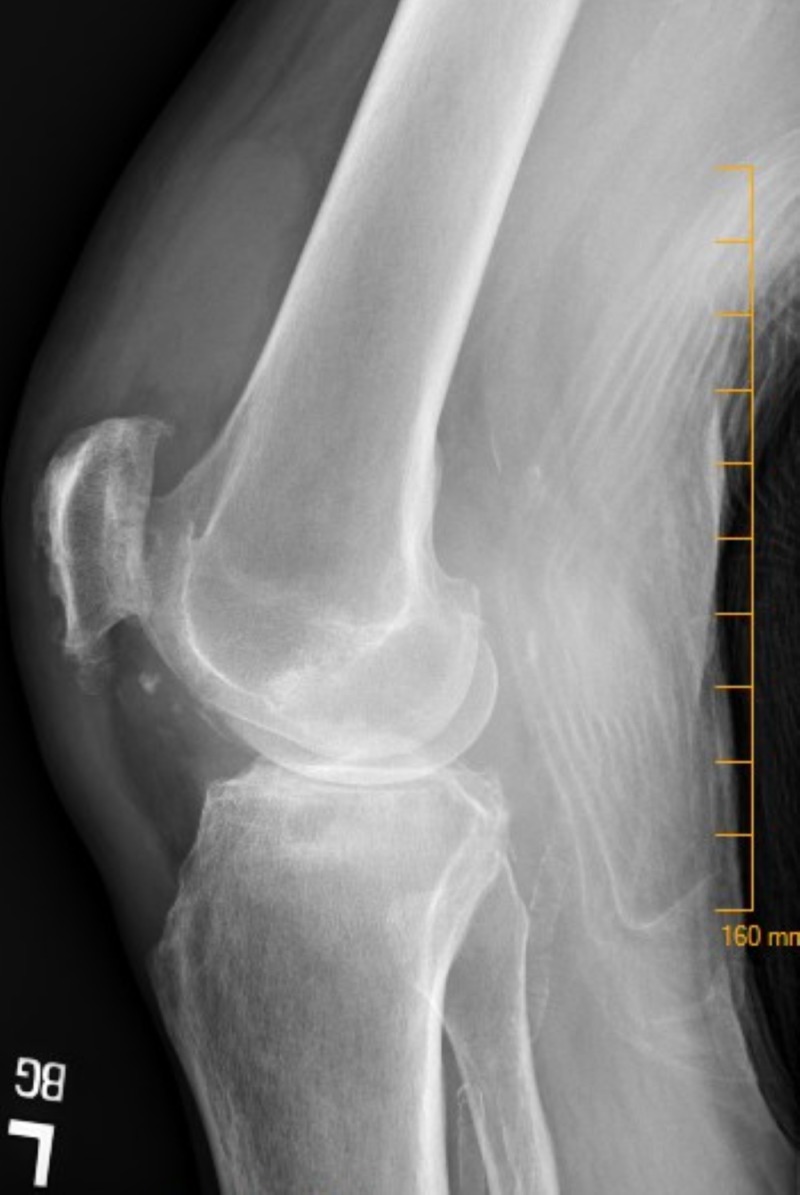
Left Knee The figure depicts a left knee x-ray demonstrating osteoarthritis and a substantial joint effusion.

The patient reported a history of gout three years prior in his right ankle and stated that his knee pain felt like his previous gout attacks. During the week preceding his ER visit, the patient was prescribed colchicine and subsequently a Medrol dose pack, but the patient experienced minimal relief in symptoms. He denied ever developing fever or chills, and his temperature remained within normal limits during his ER visit. On exam, his left knee was swollen, tender to palpation, warm to touch, and he was unable to bear weight. X-ray of the left knee demonstrated severe osteoarthritis with a significant joint effusion (Figure 3).

The ER physician aspirated 15 mL of yellowish-pink drainage for culture. The knee was injected with 1% lidocaine and triamcinolone for symptom control. Ultimately, the patient went home with a diagnosis of gout and a new prescription for clindamycin 300 mg four times a day for 10 days. The aspirated synovial fluid demonstrated leukocytosis with 94% polymorphonuclear leucocytes and uric acid crystals. Gram stain initially revealed no organisms, but eventually cultures grew MRSA. The team subsequently admitted him for IV antibiotics.

On hospital admit, his temperature remained within normal range with elevated serum uric acid levels and white blood cell counts. Gout now affected multiple joints. Examination of the left leg revealed warmth, tenderness to palpation, and an effusion with one plus pitting edema. His right shoulder had an effusion with limited range of motion. His right hand demonstrated tenderness, pain, and swelling of the first phalanx and third phalanx at the proximal interphalangeal joints and metacarpophalangeal joints. Additionally, his left wrist had pain and tenderness on rotation. 

Orthopedics aspirated his left knee and sent fluid aspirate for culture, which returned positive for MRSA. The team also washed out the knee with normal saline for two days, and then performed a left knee arthroscopic irrigation and debridement in the operating room (OR). Findings in the OR included inflamed synovium, chondral loose body, and fibrinous debris. The surgeon collected new cultures, which returned negative. He received Depo-Medrol 20 mg injections in bilateral shoulders, Depo-Medrol 40 mg injection in his left knee, and Depo-Medrol 80 mg intramuscularly. Additionally, he received colchicine 0.3 mg daily and allopurinol 100 mg daily for gout management. 

**Figure 4:**
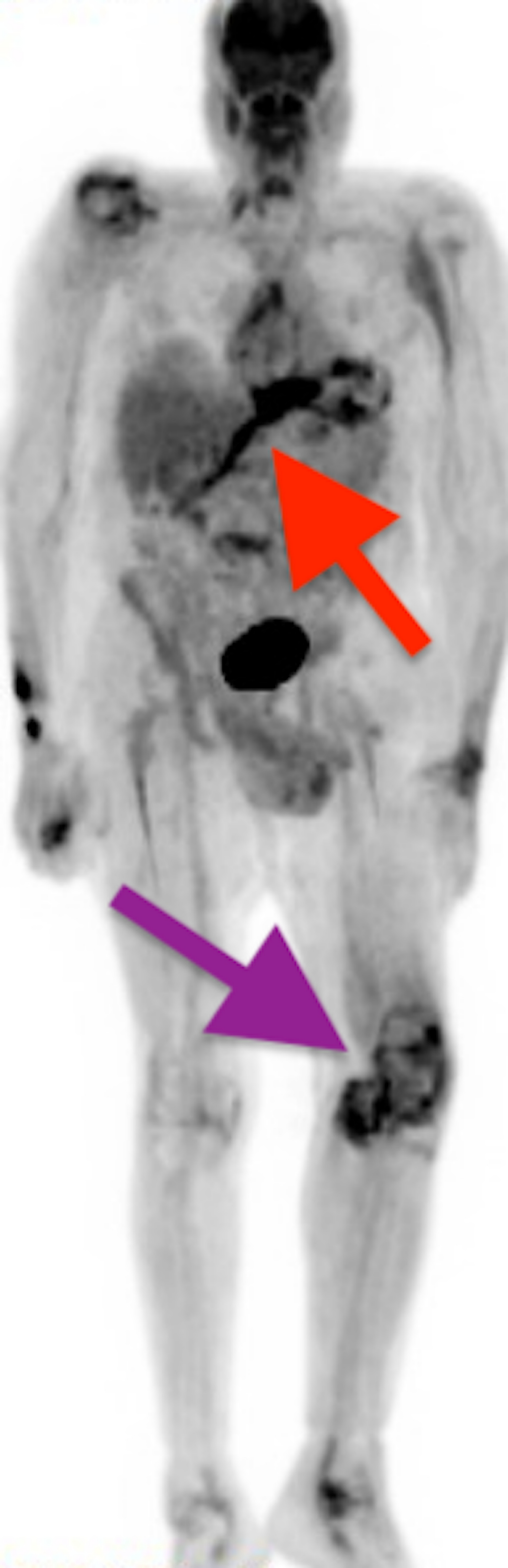
Positron Emission Tomography (PET) Scan The figure depicts a full body PET scan demonstrating hypermetabolic activity distal to the pump (red arrow) and increased metabolic activity in the left knee (purple arrow).

On admission, the team obtained blood cultures which returned positive for MRSA. The blood cultures were positive for two days with 2/2 on the first day and 1/2 on the second day. Simultaneously, he had a positron emission tomography) scan demonstrating increased metabolic activity in the thoracic cavity suggesting infection along the driveline (Figure 4).

Initially, the patient was started on IV vancomycin 1.75 g every 24 hours based on a creatinine clearance of 43.2 mL/min. Infectious disease was consulted and recommended transitioning to IV ceftaroline 600 mg every 12 hours and rifampin 300 mg by mouth every eight hours. While completing a six-week course of rifampin, the patient underwent extensive inpatient physical rehab to improve range of motion and strength. After a 78-day hospital admission, he went home on long-term ceftaroline 600 mg every 12 hours for suppression. 

## Discussion

We present a patient with a percutaneous driveline infection that remained controlled with suppressive antibiotics for approximately five months. The cardiothoracic team monitored suppression through repeat cultures that confirmed appropriate antibiotics, a CT scan, and complete blood counts. The percutaneous infection progressed to bacteremia while the patient experienced a gout attack which led to seeding of the joint and resulted in septic arthritis. Unfortunately, his gout attack initially masked his sepsis presentation. Due to the patient's medical history of gout, his primary care provider (PCP) initially prescribed colchicine for the treatment of an acute gout flare. When that failed, his physician prescribed a Medrol dose pack. Nevertheless, the attack also failed steroid treatment, and he presented to the ER. The ER doctor collected a synovial fluid culture, but providers were still not heavily contemplating a diagnosis of septic arthritis. The culture came back several days later and provided a diagnosis of septic arthritis. Thus, 18 days lapsed between the onset of symptoms and the diagnosis of sepsis. This led our team to further explore the relationship between gout and septic arthritis. 

Gout is an inflammatory arthritis induced by crystal deposition in the joints secondary to an increase in uric acid concentration. Crystal deposits tend to develop at cooler joints, such as the knees and elbows, and the joint will become warm, edematous, painful, and red [5,6]. Common modalities for the treatment include non-steroidal anti-inflammatory drugs, colchicine, steroids, allopurinol, and febuxostat [7]. Most gout attacks will resolve within a few days to two weeks, and the patient will remain asymptomatic between attacks. Initial diagnosis is based on the presence of monosodium urate crystals in the synovial fluid of the affected joint [5]. Thereafter, it is unnecessary to repeat aspiration unless the joint appears infected. The crux of the matter is that both gout and septic arthritis induce similar symptoms, which make it difficult to differentiate or identify coexistence. Frequent warning signs for both conditions include joints that are swollen, erythematous, warm to the touch, and possibly fever [4]. Knee joints are frequently affected, and Staphylococcus aureus is one of leading pathogens with an occurrence rate of 71.4% [4,6]. Septic arthritis is also more common in patients with gout, diabetes, and chronic kidney disease [4,8]. It occurs when a patient develops bacteremia, which then metastasizes to joints containing intra-articular crystals leading to seeding of the joint [3,4]. The conclusive diagnosis is based on sodium biurate crystals in the synovial joint, bacterial cultures, and gram stain [4,8]. The synovial fluid may also be cloudy with a high leukocyte count [8]. 

Our patient's case represents the difficulties we face in differentiating between gout and septic arthritis. Gout initially presented in the classic fashion and affected the knee. He had a history of gout, and uric acid levels were slightly elevated about a week before he reported symptoms. His PCP prescribed colchicine, which is standard of care. When it failed, he took a Medrol dose pack, which is also an appropriate medication. However, steroids also failed, and he presented to the ER where a culture confirmed septic arthritis. Our patient had pre-existing conditions, such as gout and chronic kidney disease that predisposed him to developing septic arthritis. He also had a known MRSA infection, which is one of the leading pathogens for septic arthritis. Our treatment of the septic joint mirrored standard of care. The joint was aspirated for culture, and then lavaged and debrided [4,8]. He was also started on appropriate antibiotics based on the culture report as well as anti-inflammatory agents and medications to reduce serum uric acid levels [8]. 

Our patient initially had an MRSA percutaneous driveline infection, and the occurrence rate for this type of infection is 14%-19% per a patient year [1,2,9]. Patients with subcutaneous driveline infections often present with localized erythema, heat, drainage, bleeding, or pain at the site, and diagnosis is confirmed by culture. Culture is typically positive for bacteria, the most common of which is S. aureus. Standard treatment for percutaneous infections includes antibiotics and in some cases debridement [1,2]. These localized infections progress to sepsis at a rate of 17.2%-18% in the infected population, and the development of a biofilm on the driveline is thought to contribute to infection development and progression [1,2,9]. Sepsis is an inflammatory response to infection in the body, commonly associated with positive blood cultures and hypotension [1,2]. From our experience, septic patients typically present with systemic symptoms of infection including fever, chills, nausea, vomiting, and symptoms at the driveline exit site. Our patient presented with typical symptoms of a percutaneous driveline infection. He developed soreness at the driveline exit site and reported a small amount of dried blood during dressing changes at home. He was examined in clinic, and a horizontal 9.5 × 3 mm line of erythema was noted with thick, purulent drainage. Culture reports confirmed MRSA and guided antibiotic treatment. Despite appropriate antibiotics, the infection progressed to sepsis. However, the patient did not present in the typical systemic fashion. His presentation was unusual in that sepsis was identified only after he presented with septic arthritis, which led to the collection of blood cultures. 

In this case report, we present a patient with a known MRSA driveline infection progressing to sepsis in an atypical fashion. At the time his driveline infection progressed to bacteremia, he was simultaneously developing a gout flare up. The gout flare produced uric acid crystals, edema, and inflammation, which led to seeding of the joint [3]. A positive knee aspirate culture was the first clue that he had progressed to sepsis, and this was collected after two failed gout treatments. In retrospect, it would have been wise to consider culturing knee aspirate after the first failed therapy. 

## Conclusions

The patient’s presentation of sepsis is unusual and caused difficulties in diagnosis and management. This case illustrates the importance of closely monitoring for progression of percutaneous infections. Our team would recommend giving strong consideration to performing joint aspiration in a patient with a driveline infection and history of gout after one treatment failure. Furthermore, this case accentuates the need for future research on the relationship between sepsis and gout. Early diagnosis combined with aggressive treatment promotes a more favorable outcome. 
